# Supplementary material for: SARS-CoV-2 Delta Variant Displays Moderate Resistance to Neutralizing Antibodies and Spike Protein Properties of Higher Soluble ACE2 Sensitivity, Enhanced Cleavage and Fusogenic Activity
Source: Viruses. 2021 Dec 11;13(12):2485. doi: 10.3390/v13122485 (PMC8707919; doi:10.3390/v13122485)
Supplement: Supplementary file 1 [file viruses-13-02485-s001.zip › viruses-1461970-supplementary.pdf]

# Supplementary Figure S1

**A**

```
Original        ATGTTTGTTTTTCTTGTTTTATTGCCACTAGTCTCTAGTCAGTGTGTTAATCTTACAACC      60
Optimized       ATGTTCGTGTTTCTGGTGCTGCTGCCTCTGGTGAGCTCCCAGTGCGTGAACCTGACCACA      60
                ***** ** ***** **  *  **** ** **       ***** ** ** ** ** **

Original        AGAACTCAATTACCCCCTGCATACACTAATTCTTTCACACGTGGTGTTTATTACCCTGAC      120
Optimized       AGGACCCAGCTGCCCCCTGCCTATACCAATTCCTTCACACGGGGCGTGTACTATCCCGAC      120
                ** ** **  * ******** ** ***** ******** ** ** ** ** ** ***

Original        AAAGTTTTCAGATCCTCAGTTTTACATTCAACTCAGGACTTGTTCTTACCTTTCTTTTCC      180
Optimized       AAGGTGTTTAGATCTAGCGTGCTGCACTCCACACAGGATCTGTTTCTGCCTTTCTTTTCT      180
                ** ** ** *****    **  * ** ** ** *****  ****  * ***********

Original        AATGTTACTTGGTTCCATGCTATACATGTCTCTGGGACCAATGGTACTAAGAGGTTTGAT      240
Optimized       AACGTGACCTGGTTCCACGCCATCCACGTGAGCGGCACCAATGGCACAAAGCGGTTCGAC      240
                ** ** ** ******** ** ** ** **    ** ******** ** *** **** **

Original        AACCCTGTCCTACCATTTAATGATGGTGTTTATTTTGCTTCCACTGAGAAGTCTAACATA      300
Optimized       AATCCAGTGCTGCCCTTTAACGATGGCGTGTACTTCGCCTCCACCGAGAAGTCTAACATC      300
                ** ** ** ** ** ***** ***** ** ** ** ** ***** **************

Original        ATAAGAGGCTGGATTTTTGGTACTACTTTAGATTCGAAGACCCAGTCCCTACTTATTGTT      360
Optimized       ATCAGAGGCTGGATCTTTGGCACCACACTGGACAGCAAGACACAGTCCCTGCTGATCGTG      360
                ** *********** ***** ** **  * **    ***** ******** ** ** **

Original        AATAACGCTACTAATGTTGTTATTAAAGTCTGTGAATTTCAATTTTGTAATGATCCATTT      420
Optimized       AACAATGCCACCAACGTGGTCATCAAGGTGTGCGAGTTCCAGTTTTGTAATGATCCATTC      420
                ** ** ** ** ** ** ** ** ** ** ** ** ** ** *****************

Original        TTGGGTGTTTATTACCACAAAAACAACAAAAGTTGGATGGAAAGTGAGTTCAGAGTTTAT      480
Optimized       CTGGGCGTGTACTATCACAAGAACAATAAGTCTTGGATGGAGAGCGAGTTTCGCGTGTAT      480
                 **** ** ** ** ***** ***** **   ********* ** *****  * ** ***

Original        TCTAGTGCGAATAATTGCACTTTTGAATATGTCTCTCAGCCTTTTCTTATGGACCTTGAA      540
Optimized       TCCTCTGCCAACAATTGCACATTTGAGTACGTGTCCCAGCCCTTCCTGATGGACCTGGAG      540
                **   *** ** ******** ***** ** ** ** ***** ** ** ******** **

Original        GGAAAACAGGGTAATTTCAAAAATCTTAGGGAATTTGTGTTTAAGAATATTGATGGTTAT      600
Optimized       GGCAAGCAGGGCAATTTCAAGAACCTGAGGGAGTTCGTGTTTAAGAATATCGATGGCTAC      600
                ** ** ***** ******** ** ** ***** ** ************** ***** **

Original        TTTAAAATATATTCTAAGCACACGCCTATTAATTTAGTGCGTGATCTCCCTCAGGGTTTT      660
Optimized       TTCAAGATCTACTCCAAGCACACCCCAATCAACCTGGTGCGCGACCTGCCACAGGGCTTC      660
                ** ** ** ** ** ******** ** ** **  * ***** ** ** ** ***** **

Original        TCGGCTTTAGAACCATTGGTAGATTTGCCAATAGGTATTAACATCACTAGGTTTCAAACT      720
Optimized       TCTGCCCTGGAGCCACTGGTGGATCTGCCCATCGGCATCAACATCACCCGGTTTCAGACA      720
                ** **  * ** *** **** *** **** ** ** ** ********  ******* **

Original        TTACTTGCTTTACATAGAAGTTATTTGACTCCTGGTGATTCTTCTTCAGGTTGGACAGCT      780
Optimized       CTGCTGGCCCTGCACAGAAGCTACCTGACACCAGGCGACAGCTCCTCTGGATGGACCGCA      780
                 * ** **  * ** ***** **  **** ** ** **    ** ** ** ***** **

Original        GGTGCTGCAGCTTATTATGTGGGTTATCTTCAACCTAGGACTTTTCTATTAAAATATAAT      840
Optimized       GGAGCAGCAGCCTACTATGTGGGCTATCTGCAGCCCAGGACCTTCCTGCTGAAGTACAAC      840
                ** ** ***** ** ******** ***** ** ** ***** ** **  * ** ** **

Original        GAAAATGGAACCATTACAGATGCTGTAGACTGTGCACTTGACCCTCTCTCAGAAACAAAG      900
Optimized       GAGAATGGCACCATCACAGACGCAGTGGATTGCGCACTGGACCCCCTGTCTGAGACCAAG      900
                ** ***** ***** ***** ** ** ** ** ***** ***** ** ** ** ** ***

Original        TGTACGTTGAAATCCTTCACTGTAGAAAAAGGAATCTATCAAACTTCTAACTTTAGAGTC      960
Optimized       TGTACACTGAAGAGCTTTACCGTGGAGAAGGGCATCTATCAGACAAGCAATTTCAGGGTG      960
                *****  ****   *** ** ** ** ** ** ******** **    ** ** ** **

Original        CAACCAACAGAATCTATTGTTAGATTTCCTAATATTACAAACTTGTGCCCTTTTGGTGAA      1020
Optimized       CAGCCTACCGAGTCCATCGTGCGCTTTCCCAATATCACAAACCTGTGCCCTTTTGGCGAG      1020
                ** ** ** ** ** ** **  * ***** ***** ****** ************* **

Original        GTTTTTAACGCCACCAGATTTGCATCTGTTTATGCTTGGAACAGGAAGAGAATCAGCAAC      1080
Optimized       GTGTTCAACGCAACCAGGTTCGCAAGCGTGTACGCATGGAATAGGAAGCGCATCTCCAAC      1080
                ** ** ***** ***** ** ***    ** ** ** ***** ****** * ***  ****

Original        TGTGTTGCTGATTATTCTGTCCTATATAATTCCGCATCATTTTCCACTTTTAAGTGTTAT      1140
Optimized       TGCGTGGCCGACTATTCTGTGCTGTACAACAGCGCCTCCTTCTCTACCTTTAAGTGCTAT      1140
                ** ** ** ** ******** ** ** **   *** ** ** ** ** ******** ***

Original        GGAGTGTCTCCTACTAAATTAAATGATCTCTGCTTTACTAATGTCTATGCAGATTCATTT      1200
Optimized       GGCGTGAGCCCCACAAAGCTGAATGACCTGTGCTTTACCAACGTGTACGCCGATTCCTTC      1200
                ** ***   ** ** **  * ***** ** ******** ** ** ** ** ***** **

Original        GTAATTAGAGGTGATGAAGTCAGACAAATCGCTCCAGGGCAAACTGGAAAGATTGCTGAT      1260
Optimized       GTGATCAGGGGCGACGAGGTGCGCCAGATCGCACCAGGACAGACAGGCAAGATCGCAGAC      1260
                ** ** ** ** ** ** **  * ** ***** ***** ** ** ** ***** ** **

Original        TATAATTATAAATTACCAGATGATTTTACAGGCTGCGTTATAGCTTGGAATTCTAACAAT      1320
Optimized       TACAATTATAAGCTGCCTGACGATTTCACCGGCTGCGTGATCGCCTGGAACTCTAACAAT      1320
                ** ********  * ** ** ***** ** ******** ** ** ***** *********

Original        CTTGATTCTAAGGTTGGTGGTAATTATAATTACCTGTATAGATTGTTTAGGAAGTCTAAT      1380
Optimized       CTGGATAGCAAAGTGGGCGGCAACTACAATTATCTGTACCGGCTGTTTAGAAAGTCTAAT      1380
                ** ***   ** ** ** ** ** ** ***** *****  *  ******* *********

Original        CTCAAACCTTTTGAGAGAGATATTTCAACTGAAATCTATCAGGCCGGTAGCACACCTTGT      1440
Optimized       CTGAAGCCATTCGAGAGGGACATCTCCACAGAGATCTACCAGGCCGGCTCTACCCCCTGC      1440
                ** ** ** ** ***** ** ** ** ** ***** ********    ** ** **

Original        AATGGTGTTGAAGGTTTTAATTGTTACTTTCCTTTACAATCATATGGTTTCCAACCCACT      1500
Optimized       AATGGCGTGGAGGGCTTTAACTGTTATTTCCCTCTGCAGAGCTACGGCTTCCAGCCAACA      1500
                ***** ** ** ** ***** ***** ** *** * **    ** ** ***** ** **

Original        AATGGTGTTGGTTACCAACCATACAGAGTAGTAGTACTTTCTTTTGAACTTCTACATGCA      1560
Optimized       AACGGCGTGGGCTATCAGCCCTACCGCGTGGTGGTGCTGTCTTTTGAGCTGCTGCACGCA      1560
                ** ** ** ** ** ** ** *** * ** ** ** ** ******** ** ** ** ***

Original        CCAGCAACTGTTTGTGGACCTAAAAAGTCTACTAATTTGGTTAAAAACAAATGTGTCAAT      1620
Optimized       CCTGCAACAGTGTGCGGACCAAAGAAGAGCACCAATCTGGTGAAGAACAAGTGCGTGAAC      1620
                ** ***** ** ** ***** ** ***   ** *** **** ** ***** ** ** **

Original        TTCAACTTCAATGGTTTAACAGGCACAGGTGTTCTTACTGAGTCTAACAAAAAGTTTCTG      1680
Optimized       TTCAACTTCAACGGACTGACCGGCACAGGCGTGCTGACCGAGTCCAACAAGAAGTTCCTG      1680
                *********** **  * ** ******** ** ** ** ***** ***** ***** ***

Original        CCTTTCCAACAATTTGGCAGAGACATTGCTGACACTACTGATGCTGTCCGTGATCCACAG      1740
Optimized       CCTTTTCAGCAGTTCGGCAGGGACATCGCAGATACCACAGACGCCGTGCGCGACCCTCAG      1740
                ***** ** ** ** ***** ***** ** ** ** ** ** ** ** ** ** ** ***

Original        ACACTTGAGATTCTTGACATTACACCATGTTCTTTTGGTGGTGTCAGTGTTATAACACCA      1800
Optimized       ACCCTGGAGATCCTGGATATCACACCATGCTCCTTCGGCGGCGTGTCTGTGATCACACCA      1800
                ** ** ***** ** ** ** ******** ** ** ** ** **   *** ** ******

Original        GGAACAAATACTTCTAACCAGGTTGCTGTTCTTTATCAGGATGTTAACTGCACAGAAGTC      1860
Optimized       GGCACCAATACAAGCAACCAGGTGGCCGTGCTGTATCAGGACGTGAATTGTACCGAGGTG      1860
                ** ** *****    ******** ** ** ** ******** ** ** ** ** ** **

Original        CCTGTTGCTATTCATGCAGATCAACTTACTCCTACTTGGCGTGTTTATTCTACAGGTTCT      1920
Optimized       CCCGTGGCAATCCACGCAGATCAGCTGACCCCTACATGGCGGGTGTACTCTACCGGCAGC      1920
                ** ** ** ** ** ******** ** ** ***** ***** ** ** ***** **

Original        AATGTTTTTCAAACACGTGCAGGCTGTTTAATAGGGGCTGAACATGTCAACAACTCATAT      1980
Optimized       AACGTGTTCCAGACAAGAGCAGGATGCCTGATCGGAGCAGAGCACGTGAACAATAGCTAT      1980
                ** ** ** ** *** * ***** **  * ** ** ** ** ** ** *****    ***

Original        GAGTGTGACATACCCATTGGTGCAGGTATATGCGCTAGTTATCAGACTCAGACTAATTCT      2040
Optimized       GAGTGCGACATCCCTATCGGCGCCGGCATCTGTGCCTCCTACCAGACCCAGACAAACTCC      2040
                ***** ***** ** ** ** ** ** ** ** **    ** ***** ***** ** **
```

```
Original      CCTCGGCGGGCACGTAGTGTAGCTAGTCAATCCATCATTGCCTACACTATGTCACTTGGT     2100
Optimized     CCAAGGAGAGCACGGTCTGTGGCAAGCCAGTCCATCATCGCCTATACCATGAGCCTGGGC     2100
              **  ** * *****   *** ** ** ** ******** ***** ** ***   ** **

Original      GCAGAAAATTCAGTTGCTTACTCTAATAACTCTATTGCCATACCCACAAATTTTACTATT     2160
Optimized     GCCGAGAATTCCGTGGCCTACTCCAACAATTCTATCGCCATCCCTACCAACTTCACAATC     2160
              ** ** ***** ** ** ***** ** ** ***** ***** ** ** ** ** ** **

Original      AGTGTTACCACAGAAATTCTACCAGTGTCTATGACCAAGACATCAGTAGATTGTACAATG     2220
Optimized     TCCGTGACCACAGAGATCCTGCCAGTGAGCATGACCAAGACATCCGTGGACTGCACAATG     2220
                 ** ******** ** ** ******   ************** ** ** ** ******

Original      TACATTTGTGGTGATTCAACTGAATGCAGCAATCTTTTGTTGCAATATGGCAGTTTTTGT     2280
Optimized     TATATCTGTGGCGATTCCACCGAGTGCTCTAACCTGCTGCTGCAGTACGGCTCTTTTTGT     2280
              ** ** ***** ***** ** ** ***   ** **  ** **** ** ***  *******

Original      ACACAATTAAACCGTGCTTTAACTGGAATAGCTGTTGAACAAGACAAAAACACCCAAGAA     2340
Optimized     ACCCAGCTGAATAGAGCCCTGACAGGCATCGCCGTGGAGCAGGACAAGAACACACAGGAG     2340
              ** **  * **  * **  * ** ** ** ** ** ** ** ***** ***** ** **

Original      GTTTTTGCACAAGTCAAACAAATTTACAAAACACCACCAATTAAAGATTTTGGTGGTTTT     2400
Optimized     GTGTTCGCCCAGGTGAAGCAGATCTACAAGACCCCACCCATCAAGGACTTTGGCGGCTTC     2400
              ** ** ** ** ** ** ** ** ***** ** ***** ** ** ** ***** ** **

Original      AATTTTTCACAAATATTACCAGATCCATCAAAACCAAGCAAGAGGTCATTTATTGAAGAT     2460
Optimized     AACTTCAGCCAGATCCTGCCCGATCCTAGCAAGCCATCCAAGCGGTCTTTTATCGAGGAC     2460
              ** **    ** **  * ** *****    ** ***  **** **** ***** ** **

Original      CTACTTTTCAACAAAGTGACACTTGCAGATGCTGGCTTCATCAAACAATATGGTGATTGC     2520
Optimized     CTGCTGTTCAACAAGGTGACCCTGGCCGATGCCGGCTTCATCAAGCAGTATGGCGATTGC     2520
              ** ** ******** ***** ** ** ***** *********** ** ***** ******

Original      CTTGGTGATATTGCTGCTAGAGACCTCATTTGTGCACAAAAGTTTAACGGCCTTACTGTT     2580
Optimized     CTGGGCGACATCGCCGCCAGAGACCTGATCTGTGCCCAGAAGTTTAATGGCCTGACCGTG     2580
              ** ** ** ** ** ** ******** ** ***** ** ******** ***** ** **

Original      TTGCCACCTTTGCTCACAGATGAAATGATTGCTCAATACACTTCTGCACTGTTAGCGGGT     2640
Optimized     CTGCCTCCACTGCTGACAGATGAGATGATCGCCCAGTACACATCTGCCCTGCTGGCAGGC     2640
               **** **  **** ******** ***** ** ** ***** ***** *** * ** **

Original      ACAATCACTTCTGGTTGGACCTTTGGTGCAGGTGCTGCATTACAAATACCATTTGCTATG     2700
Optimized     ACCATCACAAGCGGATGGACCTTCGGCGCAGGAGCCGCCCTGCAGATCCCCTTTGCCATG     2700
              ** *****    ** ******** ** ***** ** **  * ** ** ** ***** ***

Original      CAAATGGCTTATAGGTTTAATGGTATTGGAGTTACACAGAATGTTCTCTATGAGAACCAA     2760
Optimized     CAGATGGCCTATCGGTTCAACGGCATCGGCGTGACCCAGAATGTGCTGTACGAGAACCAG     2760
              ** ***** *** **** ** ** ** ** ** ** ******** ** ** ********

Original      AAATTGATTGCCAACCAATTTAATAGTGCTATTGGCAAAATTCAAGACTCACTTTCTTCC     2820
Optimized     AAGCTGATCGCCAATCAGTTTAACTCCGCCATCGGCAAGATCCAGGACTCTCTGAGCTCC     2820
              **  **** ***** ** *****    ** ** ***** ** ** ***** **    ***

Original      ACAGCAAGTGCACTTGGAAAACTTCAAGATGTGGTCAACCAAAATGCACAAGCTTTAAAC     2880
Optimized     ACAGCAAGCGCCCTGGGCAAGCTGCAGGATGTGGTGAATCAGAACGCCCAGGCCCTGAAT     2880
              ******** ** ** ** ** ** ** ******** ** ** ** ** ** **  * **

Original      ACGCTTGTTAAACAACTTAGCTCCAATTTTGGTGCAATTTCAAGTGTTTTAAATGATATC     2940
Optimized     ACCCTGGTGAAGCAGCTGTCTAGCAACTTCGGCGCCATCTCCTCTGTGCTGAATGATATC     2940
              ** ** ** ** ** **      *** ** ** ** ** **   ***  * *********

Original      CTTTCACGTCTTGACAAAGTTGAGGCTGAAGTGCAAATTGATAGGTTGATCACAGGCAGA     3000
Optimized     CTGAGCAGGCTGGACAAGGTGGAGGCAGAGGTGCAGATCGACCGGCTGATCACAGGCAGA     3000
              **     * ** ***** ** ***** ** ***** ** **  ** **************

Original      CTTCAAAGTTTGCAGACATATGTGACTCAACAATTAATTAGAGCTGCAGAAATCAGAGCT     3060
Optimized     CTGCAGTCCCTGCAGACCTACGTGACACAGCAGCTGATCAGGGCAGCAGAGATCAGGGCA     3060
              ** **     ******* ** ***** ** **  * ** ** ** ***** ***** **

Original      TCTGCTAATCTTGCTGCTACTAAAATGTCAGAGTGTGTACTTGGACAATCAAAAAGAGTT     3120
Optimized     TCTGCCAATCTGGCCGCCACCAAGATGAGCGAGTGCGTGCTGGGCCAGTCCAAGAGAGTG     3120
              ***** ***** ** ** ** ** ***   ***** ** ** ** ** ** ** *****

Original      GATTTTTGTGGAAAGGGCTATCATCTTATGTCCTTCCCTCAGTCAGCACCTCATGGTGTA     3180
Optimized     GACTTTTGTGGCAAGGGCTATCACCTGATGAGCTTCCCACAGTCCGCCCCTCACGGAGTG     3180
              ** ******** *********** ** ***  ****** ***** ** ***** ** **

Original      GTCTTCTTGCATGTGACTTATGTCCCTGCACAAGAAAAGAACTTCACAACTGCTCCTGCC     3240
Optimized     GTGTTTCTGCACGTGACCTACGTGCCAGCCCAGGAGAAGAACTTCACCACAGCACCAGCA     3240
              ** **  **** ***** ** ** ** ** ** ** *********** ** ** ** **

Original      ATTTGTCATGATGGAAAAGCACACTTTCCTCGTGAAGGTGTCTTTGTTTCAAATGGCACA     3300
Optimized     ATCTGCCACGATGGCAAGGCACACTTTCCTAGGGAGGGCGTGTTCGTGAGCAACGGCACC     3300
              ** ** ** ***** ** ************ * ** ** ** ** **    ** *****

Original      CACTGGTTTGTAACACAAAGGAATTTTTATGAACCACAAATCATTACTACAGACAACACA     3360
Optimized     CACTGGTTTGTGACACAGCGCAATTTCTACGAGCCACAGATCATCACCACAGACAATACA     3360
              *********** *****  * ***** ** ** ***** ***** ** ******** ***

Original      TTTGTGTCTGGTAACTGTGATGTTGTAATAGGAATTGTCAACAACACAGTTTATGATCCT     3420
Optimized     TTCGTGTCCGGCAACTGTGACGTGGTCATCGGCATCGTGAACAATACCGTGTATGATCCT     3420
              ** ***** ** ******** ** ** ** ** ** ***** ** ** *********

Original      TTGCAACCTGAATTAGACTCATTCAAGGAGGAGTTAGATAAATATTTTAAGAATCATACA     3480
Optimized     CTGCAGCCAGAGCTGGACTCTTTTAAGGAGGAGCTGGATAAGTACTTCAAGAATCACACC     3480
               **** ** **  * ***** ** ********* * ***** ** ** ******** **

Original      TCACCAGATGTTGATTTAGGTGACATCTCTGGCATTAATGCTTCAGTTGTAAACATTCAA     3540
Optimized     AGCCCCGACGTGGATCTGGGCGACATCTCTGGCATCAATGCCAGCGTGGTGAACATCCAG     3540
                 ** ** ** *** * ** ************** *****    ** ** ***** **

Original      AAAGAAATTGACCGCCTCAATGAGGTTGCCAAGAATTTAAATGAATCTCTCATCGATCTC     3600
Optimized     AAGGAGATCGACAGGCTGAACGAGGTGGCCAAGAATCTGAACGAGTCCCTGATCGATCTG     3600
              ** ** ** *** * ** ** ***** ********* * ** ** ** ** ********

Original      CAAGAACTTGGAAAGTATGAGCAGTATATAAAATGGCCATGGTACATTTGGCTAGGTTTT     3660
Optimized     CAGGAGCTGGGCAAGTATGAGCAGTACATCAAGTGGCCCTGGTATATCTGGCTGGGCTTC     3660
              ** ** ** ** ************** ** ** ***** ***** ** ***** ** **

Original      ATAGCTGGCTTGATTGCCATAGTAATGGTGACAATTATGCTTTGCTGTATGACCAGTTGC     3720
Optimized     ATCGCCGGCCTGATCGCCATCGTGATGGTGACCATCATGCTGTGCTGTATGACAAGCTGC     3720
              ** ** *** **** ***** ** ******** ** ***** *********** ** ***

Original      TGTAGTTGTCTCAAGGGCTGTTGTTCTTGTGGATCCTGCTGCAAATTTGATGAAGACGAC     3780
Optimized     TGTTCCTGCCTGAAGGGCTGCTGTTCTTGTGGCAGCTGCTGTAAGTTTGATGAGGACGAT     3780
              ***   ** ** ******** ***********   ****** ** ******** *****

Original      TCTGAGCCAGTGCTCAAAGGAGTCAAATTACATTACACATAA       3822
Optimized     AGCGAGCCTGTGCTGAAGGGCGTGAAGCTGCACTACACCTAA       3822
                 ***** ***** ** ** ** **  * ** ***** ***…
```

**B**

```
Pfizer         ATGTTCGTGTTCCTGGTGCTGCTGCCTCTGGTGTCCAGCCAGTGTGTGAACCTGACCACC    60
Moderna        ATGTTCGTGTTCCTGGTGCTGCTGCCCCTGGTGAGCAGCCAGTGCGTGAACCTGACCACC    60
Optimized      ATGTTCGTGTTTCTGGTGCTGCTGCCTCTGGTGAGCTCCCAGTGCGTGAACCTGACCACA    60
               *********** ************** ******  *  ****** **************

Pfizer         AGAACACAGCTGCCTCCAGCCTACACCAACAGCTTTACCAGAGGCGTGTACTACCCCGAC    120
Moderna        CGGACCCAGCTGCCACCAGCCTACACCAACAGCTTCACCCGGGGCGTCTACTACCCCGAC    120
Optimized      AGGACCCAGCTGCCCCCTGCCTATACCAATTCCTTCACACGGGGCGTGTACTATCCCGAC    120
                * ** ******** ** ***** *****   *** **  * ***** ***** ******

Pfizer         AAGGTGTTCAGATCCAGCGTGCTGCACTCTACCCAGGACCTGTTCCTGCCTTTCTTCAGC    180
Moderna        AAGGTGTTCCGGAGCAGCGTCCTGCACAGCACCCAGGACCTGTTCCTGCCCTTCTTCAGC    180
Optimized      AAGGTGTTTAGATCTAGCGTGCTGCACTCCACACAGGATCTGTTTCTGCCTTTCTTTTCT    180
               ********  *    ***** ******   ** ***** ***** ***** *****

Pfizer         AACGTGACCTGGTTCCACGCCATCCACGTGTCCGGCACCAATGGCACCAAGAGATTCGAC    240
Moderna        AACGTGACCTGGTTCCACGCCATCCACGTGAGCGGCACCAACGGCACCAAGCGGTTCGAC    240
Optimized      AACGTGACCTGGTTCCACGCCATCCACGTGAGCGGCACCAATGGCACAAAGCGGTTCGAC    240
               ******************************  ********* ***** *** * ******

Pfizer         AACCCCGTGCTGCCCTTCAACGACGGGGTGTACTTTGCCAGCACCGAGAAGTCCAACATC    300
Moderna        AACCCCGTGCTGCCCTTCAACGACGGCGTGTACTTCGCCAGCACCGAGAAGAGCAACATC    300
Optimized      AATCCAGTGCTGCCCTTTAACGATGGCGTGTACTTCGCCTCCACCGAGAAGTCTAACATC    300
               ** ** *********** ***** ** ******** ***  **********   ******

Pfizer         ATCAGAGGCTGGATCTTCGGCACCACACTGGACAGCAAGACCCAGAGCCTGCTGATCGTG    360
Moderna        ATCCGGGGCTGGATCTTCGGCACCACCCTGGACAGCAAGACCCAGAGCCTGCTGATCGTG    360
Optimized      ATCAGAGGCTGGATCTTTGGCACCACACTGGACAGCAAGACACAGTCCCTGCTGATCGTG    360
               *** * *********** ******** ************** ***  *************

Pfizer         AACAACGCCACCAACGTGGTCATCAAAGTGTGCGAGTTCCAGTTCTGCAACGACCCCTTC    420
Moderna        AATAACGCCACCAACGTGGTGATCAAGGTGTGCGAGTTCCAGTTCTGCAACGACCCCTTC    420
Optimized      AACAATGCCACCAACGTGGTCATCAAGGTGTGCGAGTTCCAGTTTTGTAATGATCCATTC    420
               ** ** ************** ***** ***************** ** ** ** ** ***

Pfizer         CTGGGCGTCTACTACCACAAGAACAACAAGAGCTGGATGGAAAGCGAGTTCCGGGTGTAC    480
Moderna        CTGGGCGTGTACTACCACAAGAACAACAAGAGCTGGATGGAGAGCGAGTTCCGGGTGTAC    480
Optimized      CTGGGCGTGTACTATCACAAGAACAATAAGTCTTGGATGGAGAGCGAGTTTCGCGTGTAT    480
               ******** ***** *********** ***   ******** ******** ** *****

Pfizer         AGCAGCGCCAACAACTGCACCTTCGAGTACGTGTCCCAGCCTTTCCTGATGGACCTGGAA    540
Moderna        AGCAGCGCCAACAACTGCACCTTCGAGTACGTGAGCCAGCCCTTCCTGATGGACCTGGAG    540
Optimized      TCCTCTGCCAACAATTGCACATTTGAGTACGTGTCCCAGCCCTTCCTGATGGACCTGGAG    540
                 *   ******** ***** ** *********  ****** *****************

Pfizer         GGCAAGCAGGGCAACTTCAAGAACCTGCGCGAGTTCGTGTTTAAGAACATCGACGGCTAC    600
Moderna        GGCAAGCAGGGCAACTTCAAGAACCTGCGGGAGTTCGTGTTCAAGAACATCGACGGCTAC    600
Optimized      GGCAAGCAGGGCAATTTCAAGAACCTGAGGGAGTTCGTGTTTAAGAATATCGATGGCTAC    600
               ************** ************ * *********** ***** ***** ******

Pfizer         TTCAAGATCTACAGCAAGCACACCCCTATCAACCTCGTGCGGGATCTGCCTCAGGGCTTC    660
Moderna        TTCAAGATCTACAGCAAGCACACCCCAATCAACCTGGTGCGGGATCTGCCCCAGGGCTTC    660
Optimized      TTCAAGATCTACTCCAAGCACACCCCAATCAACCTGGTGCGCGACCTGCCACAGGGCTTC    660
               ************  ************ ******** ***** ** ***** *********

Pfizer         TCTGCTCTGGAACCCCTGGTGGATCTGCCCATCGGCATCAACATCACCCGGTTTCAGACA    720
Moderna        TCAGCCCTGGAGCCCCTGGTGGACCTGCCCATCGGCATCAACATCACCCGGTTCCAGACC    720
Optimized      TCTGCCCTGGAGCCACTGGTGGATCTGCCCATCGGCATCAACATCACCCGGTTTCAGACA    720
               ** ** ***** ** ******** ***************************** *****

Pfizer         CTGCTGGCCCTGCACAGAAGCTACCTGACACCTGGCGATAGCAGCAGCGGATGGACAGCT    780
Moderna        CTGCTGGCCCTGCACCGGAGCTACCTGACCCCAGGCGACAGCAGCAGCGGGTGGACAGCA    780
Optimized      CTGCTGGCCCTGCACAGAAGCTACCTGACACCAGGCGACAGCTCCTCTGGATGGACCGCA    780
               *************** * *********** ** ***** ***  *   ** ***** **
```

```
Pfizer          GGTGCCGCCGCTTACTATGTGGGCTACCTGCAGCCTAGAACCTTCCTGCTGAAGTACAAC      840
Moderna         GGCGCGGCTGCTTACTACGTGGGCTACCTGCAGCCCCGGACCTTCCTGCTGAAGTACAAC      840
Optimized       GGAGCAGCAGCCTACTATGTGGGCTATCTGCAGCCCAGGACCTTCCTGCTGAAGTACAAC      840
                ** ** ** ** ***** ******** ********  * *********************

Pfizer          GAGAACGGCACCATCACCGACGCCGTGGATTGTGCTCTGGATCCTCTGAGCGAGACAAAG      900
Moderna         GAGAACGGCACCATCACCGACGCCGTGGACTGCGCCCTGGACCCTCTGAGCGAGACCAAG      900
Optimized       GAGAATGGCACCATCACAGACGCAGTGGATTGCGCACTGGACCCCCTGTCTGAGACCAAG      900
                ***** *********** ***** ***** ** ** ***** ** ***   ***** ***

Pfizer          TGCACCCTGAAGTCCTTCACCGTGGAAAAGGGCATCTACCAGACCAGCAACTTCCGGGTG      960
Moderna         TGCACCCTGAAGAGCTTCACCGTGGAGAAGGGCATCTACCAGACCAGCAACTTCCGGGTG      960
Optimized       TGTACACTGAAGAGCTTTACCGTGGAGAAGGGCATCTATCAGACAAGCAATTTCAGGGTG      960
                ** ** ******  *** ******** *********** ***** ***** *** *****

Pfizer          CAGCCCACCGAATCCATCGTGCGGTTCCCCAATATCACCAATCTGTGCCCCTTCGGCGAG      1020
Moderna         CAGCCCACCGAGAGCATCGTGCGGTTCCCCAACATCACCAACCTGTGCCCCTTCGGCGAG      1020
Optimized       CAGCCTACCGAGTCCATCGTGCGCTTTCCCAATATCACAAACCTGTGCCCTTTTGGCGAG      1020
                ***** *****   ********* ** ***** ***** ** ******** ** ******

Pfizer          GTGTTCAATGCCACCAGATTCGCCTCTGTGTACGCCTGGAACCGGAAGCGGATCAGCAAT      1080
Moderna         GTGTTCAACGCCACCCGGTTCGCCAGCGTGTACGCCTGGAACCGGAAGCGGATCAGCAAC      1080
Optimized       GTGTTCAACGCAACCAGGTTCGCAAGCGTGTACGCATGGAATAGGAAGCGCATCTCCAAC      1080
                ******** ** *** * *****    ******** *****  ******* ***  *** 

Pfizer          TGCGTGGCCGACTACTCCGTGCTGTACAACTCCGCCAGCTTCAGCACCTTCAAGTGCTAC      1140
Moderna         TGCGTGGCCGACTACAGCGTGCTGTACAACAGCGCCAGCTTCAGCACCTTCAAGTGCTAC      1140
Optimized       TGCGTGGCCGACTATTCTGTGCTGTACAACAGCGCCTCCTTCTCTACCTTTAAGTGCTAT      1140
                **************    ************  ****  ****   ***** ******** 

Pfizer          GGCGTGTCCCCTACCAAGCTGAACGACCTGTGCTTCACAAACGTGTACGCCGACAGCTTC      1200
Moderna         GGCGTGAGCCCCACCAAGCTGAACGACCTGTGCTTCACCAACGTGTACGCCGACAGCTTC      1200
Optimized       GGCGTGAGCCCCACAAAGCTGAATGACCTGTGCTTTACCAACGTGTACGCCGATTCCTTC      1200
                ******  *** ** ******** *********** ** **************   ****

Pfizer          GTGATCCGGGGAGATGAAGTGCGGCAGATTGCCCCTGGACAGACAGGCAAGATCGCCGAC      1260
Moderna         GTGATCCGTGGCGACGAGGTGCGGCAGATCGCACCCGGCCAGACAGGCAAGATCGCCGAC      1260
Optimized       GTGATCAGGGGCGACGAGGTGCGCCAGATCGCACCAGGACAGACAGGCAAGATCGCAGAC      1260
                ****** * ** ** ** ***** ***** ** ** ** ***************** ***

Pfizer          TACAACTACAAGCTGCCCGACGACTTCACCGGCTGTGTGATTGCCTGGAACAGCAACAAC      1320
Moderna         TACAACTACAAGCTGCCCGACGACTTCACCGGCTGCGTGATCGCCTGGAACAGCAACAAC      1320
Optimized       TACAATTATAAGCTGCCTGACGATTTCACCGGCTGCGTGATCGCCTGGAACTCTAACAAT      1320
                ***** ** ******** ***** *********** ***** *********   ***** 

Pfizer          CTGGACTCCAAAGTCGGCGGCAACTACAATTACCTGTACCGGCTGTTCCGGAAGTCCAAT      1380
Moderna         CTCGACAGCAAGGTGGGCGGCAACTACAACTACCTGTACCGGCTGTTCCGGAAGAGCAAC      1380
Optimized       CTGGATAGCAAAGTGGGCGGCAACTACAATTATCTGTACCGGCTGTTTAGAAAGTCTAAT      1380
                ** **   *** ** ************** ** **************  * ***   ** 

Pfizer          CTGAAGCCCTTCGAGCGGGACATCTCCACCGAGATCTATCAGGCCGGCAGCACCCCTTGT      1440
Moderna         CTGAAGCCCTTCGAGCGGGACATCAGCACCGAGATCTACCAAGCCGGCTCCACCCCTTGC      1440
Optimized       CTGAAGCCATTCGAGAGGGACATCTCCACAGAGATCTACCAGGCCGGCTCTACCCCCTGC      1440
                ******** ****** ********  *** ******** ** ******   ***** ** 

Pfizer          AACGGCGTGGAAGGCTTCAACTGCTACTTCCCACTGCAGTCCTACGGCTTTCAGCCCACA      1500
Moderna         AACGGCGTGGAGGGCTTCAACTGCTACTTCCCTCTGCAGAGCTACGGCTTCCAGCCCACC      1500
Optimized       AATGGCGTGGAGGGCTTTAACTGTTATTTCCCTCTGCAGAGCTACGGCTTCCAGCCAACA      1500
                ** ******** ***** ***** ** ***** ******  ********* ***** ** 

Pfizer          AATGGCGTGGGCTATCAGCCCTACAGAGTGGTGGTGCTGAGCTTCGAACTGCTGCATGCC      1560
Moderna         AACGGCGTGGGCTACCAGCCCTACCGGGTGGTGGTGCTGAGCTTCGAGCTGCTGCACGCC      1560
Optimized       AACGGCGTGGGCTATCAGCCCTACCGCGTGGTGGTGCTGTCTTTTGAGCTGCTGCACGCA      1560
                ** *********** ********* * ************   ** ** ******** ** 

Pfizer          CCTGCCACAGTGTGCGGCCCTAAGAAAAGCACCAATCTCGTGAAGAACAAATGCGTGAAC      1620
Moderna         CCAGCCACCGTGTGTGGCCCCAAGAAGAGCACCAACCTGGTGAAGAACAAGTGCGTGAAC      1620
Optimized       CCTGCAACAGTGTGCGGACCAAAGAAGAGCACCAATCTGGTGAAGAACAAGTGCGTGAAC      1620
                ** ** ** ***** ** ** ***** ******** ** *********** *********
```

```
Pfizer          TTCAACTTCAACGGCCTGACCGGCACCGGCGTGCTGACAGAGAGCAACAAGAAGTTCCTG     1680
Moderna         TTCAACTTCAACGGCCTTACCGGCACCGGCGTGCTGACCGAGAGCAACAAGAAATTCCTG     1680
Optimized       TTCAACTTCAACGGACTGACCGGCACAGGCGTGCTGACCGAGTCCAACAAGAAGTTCCTG     1680
                ************** ** ******** *********** ***  ********* ******

Pfizer          CCATTCCAGCAGTTTGGCCGGGATATCGCCGATACCACAGACGCCGTTAGAGATCCCCAG     1740
Moderna         CCCTTTCAGCAGTTCGGCCGGGACATCGCCGACACCACCGACGCTGTGCGGGATCCCCAG     1740
Optimized       CCTTTTCAGCAGTTCGGCAGGGACATCGCAGATACCACAGACGCCGTGCGCGACCCTCAG     1740
                ** ** ******** *** **** ***** ** ***** ***** **  * ** ** ***

Pfizer          ACACTGGAAATCCTGGACATCACCCCTTGCAGCTTCGGCGGAGTGTCTGTGATCACCCCT     1800
Moderna         ACCCTGGAGATCCTGGACATCACCCCTTGCAGCTTCGGCGGCGTGAGCGTGATCACCCCA     1800
Optimized       ACCCTGGAGATCCTGGATATCACACCATGCTCCTTCGGCGGCGTGTCTGTGATCACACCA     1800
                ** ***** ******** ***** ** ***  ********* ***   ******** **

Pfizer          GGCACCAACACCAGCAATCAGGTGGCAGTGCTGTACCAGGACGTGAACTGTACCGAAGTG     1860
Moderna         GGCACCAACACCAGCAACCAGGTGGCCGTGCTGTACCAGGACGTGAACTGCACCGAGGTG     1860
Optimized       GGCACCAATACAAGCAACCAGGTGGCCGTGCTGTATCAGGACGTGAATTGTACCGAGGTG     1860
                ******** ** ***** ******** ******** *********** ** ***** ***

Pfizer          CCCGTGGCCATTCACGCCGATCAGCTGACACCTACATGGCGGGTGTACTCCACCGGCAGC     1920
Moderna         CCCGTGGCCATCCACGCCGACCAGCTGACACCCACCTGGCGGGTCTACAGCACCGGCAGC     1920
Optimized       CCCGTGGCAATCCACGCAGATCAGCTGACCCCTACATGGCGGGTGTACTCTACCGGCAGC     1920
                ******** ** ***** ** ******** ** ** ******** ***   *********

Pfizer          AATGTGTTTCAGACCAGAGCCGGCTGTCTGATCGGAGCCGAGCACGTGAACAATAGCTAC     1980
Moderna         AACGTGTTCCAGACCCGGGCCGGTTGCCTGATCGGCGCCGAGCACGTGAACAACAGCTAC     1980
Optimized       AACGTGTTCCAGACAAGAGCAGGATGCCTGATCGGAGCAGAGCACGTGAACAATAGCTAT     1980
                ** ***** *****  * ** ** ** ******** ** ************** *****

Pfizer          GAGTGCGACATCCCCATCGGCGCTGGAATCTGCGCCAGCTACCAGACACAGACAAACAGC     2040
Moderna         GAGTGCGACATCCCCATCGGCGCCGGCATCTGTGCCAGCTACCAGACCCAGACCAATTCA     2040
Optimized       GAGTGCGACATCCCTATCGGCGCCGGCATCTGTGCCTCCTACCAGACCCAGACAAACTCC     2040
                ************** ******** ** ***** ***  ********* ***** **

Pfizer          CCTCGGAGAGCCAGAAGCGTGGCCAGCCAGAGCATCATTGCCTACACAATGTCTCTGGGC     2100
Moderna         CCCCGGAGGGCAAGGAGCGTGGCCAGCCAGAGCATCATCGCCTACACCATGAGCCTGGGC     2100
Optimized       CCAAGGAGAGCACGGTCTGTGGCAAGCCAGTCCATCATCGCCTATACCATGAGCCTGGGC     2100
                **  **** **  *    ***** ******  ****** ***** ** ***   ******

Pfizer          GCCGAGAACAGCGTGGCCTACTCCAACAACTCTATCGCTATCCCCACCAACTTCACCATC     2160
Moderna         GCCGAGAACAGCGTGGCCTACAGCAACAACAGCATCGCCATCCCCACCAACTTCACCATC     2160
Optimized       GCCGAGAATTCCGTGGCCTACTCCAACAATTCTATCGCCATCCCTACCAACTTCACAATC     2160
                ********   **********  ******    ***** ***** *********** ***

Pfizer          AGCGTGACCACAGAGATCCTGCCTGTGTCCATGACCAAGACCAGCGTGGACTGCACCATG     2220
Moderna         AGCGTGACCACCGAGATTCTGCCCGTGAGCATGACCAAGACCAGCGTGGACTGCACCATG     2220
Optimized       TCCGTGACCACAGAGATCCTGCCAGTGAGCATGACCAAGACATCCGTGGACTGCACAATG     2220
                  ********* ***** ***** ***  ************   ************ ***

Pfizer          TACATCTGCGGCGATTCCACCGAGTGCTCCAACCTGCTGCTGCAGTACGGCAGCTTCTGC     2280
Moderna         TACATCTGCGGCGACAGCACCGAGTGCAGCAACCTGCTGCTGCAGTACGGCAGCTTCTGC     2280
Optimized       TATATCTGTGGCGATTCCACCGAGTGCTCTAACCTGCTGCTGCAGTACGGCTCTTTTTGT     2280
                ** ***** *****   **********   *********************   ** **

Pfizer          ACCCAGCTGAATAGAGCCCTGACAGGGATCGCCGTGGAACAGGACAAGAACACCCAAGAG     2340
Moderna         ACCCAGCTGAACCGGGCCCTGACCGGCATCGCCGTGGAGCAGGACAAGAACACCCAGGAG     2340
Optimized       ACCCAGCTGAATAGAGCCCTGACAGGCATCGCCGTGGAGCAGGACAAGAACACACAGGAG     2340
                ***********  * ******** ** *********** ************** ** ***

Pfizer          GTGTTCGCCCAAGTGAAGCAGATCTACAAGACCCCTCCTATCAAGGACTTCGGCGGCTTC     2400
Moderna         GTGTTCGCCCAGGTGAAGCAGATCTACAAGACCCCTCCCATCAAGGACTTCGGCGGCTTC     2400
Optimized       GTGTTCGCCCAGGTGAAGCAGATCTACAAGACCCCACCCATCAAGGACTTTGGCGGCTTC     2400
                *********** *********************** ** *********** *********

Pfizer          AATTTCAGCCAGATTCTGCCCGATCCTAGCAAGCCCAGCAAGCGGAGCTTCATCGAGGAC     2460
Moderna         AACTTCAGCCAGATCCTGCCCGACCCCAGCAAGCCCAGCAAGCGGAGCTTCATCGAGGAC     2460
Optimized       AACTTCAGCCAGATCCTGCCCGATCCTAGCAAGCCATCCAAGCGGTCTTTTATCGAGGAC     2460
```

```
               ** *********** ******** ** ********   *******   ** *********

Pfizer         CTGCTGTTCAACAAAGTGACACTGGCCGACGCCGGCTTCATCAAGCAGTATGGCGATTGT     2520
Moderna        CTGCTGTTCAACAAGGTGACCCTAGCCGACGCCGGCTTCATCAAGCAGTACGGCGACTGC     2520
Optimized      CTGCTGTTCAACAAGGTGACCCTGGCCGATGCCGGCTTCATCAAGCAGTATGGCGATTGC     2520
               ************** ***** ** ***** ******************** ***** **

Pfizer         CTGGGCGACATTGCCGCCAGGGATCTGATTTGCGCCCAGAAGTTTAACGGACTGACAGTG     2580
Moderna        CTCGGCGACATAGCCGCCCGGGACCTGATCTGCGCCCAGAAGTTCAACGGCCTGACCGTG     2580
Optimized      CTGGGCGACATCGCCGCCAGAGACCTGATCTGTGCCCAGAAGTTTAATGGCCTGACCGTG     2580
               ** ******** ****** * ** ***** ** *********** ** ** ***** ***

Pfizer         CTGCCTCCTCTGCTGACCGATGAGATGATCGCCCAGTACACATCTGCCCTGCTGGCCGGC     2640
Moderna        CTGCCTCCCCTGCTGACCGACGAGATGATCGCCCAGTACACCAGCGCCCTGTTAGCCGGA     2640
Optimized      CTGCCTCCACTGCTGACAGATGAGATGATCGCCCAGTACACATCTGCCCTGCTGGCAGGC     2640
               ******** ******** ** ********************    ****** * ** **

Pfizer         ACAATCACAAGCGGCTGGACATTTGGAGCAGGCGCCGCTCTGCAGATCCCCTTTGCTATG     2700
Moderna        ACCATCACCAGCGGCTGGACTTTCGGCGCTGGAGCCGCTCTGCAGATCCCCTTCGCCATG     2700
Optimized      ACCATCACAAGCGGATGGACCTTCGGCGCAGGAGCCGCCCTGCAGATCCCCTTTGCCATG     2700
               ** ***** ***** ***** ** ** ** ** ***** ************** ** ***

Pfizer         CAGATGGCCTACCGGTTCAACGGCATCGGAGTGACCCAGAATGTGCTGTACGAGAACCAG     2760
Moderna        CAGATGGCCTACCGGTTCAACGGCATCGGCGTGACCCAGAACGTGCTGTACGAGAACCAG     2760
Optimized      CAGATGGCCTATCGGTTCAACGGCATCGGCGTGACCCAGAATGTGCTGTACGAGAACCAG     2760
               *********** ***************** *********** ******************

Pfizer         AAGCTGATCGCCAACCAGTTCAACAGCGCCATCGGCAAGATCCAGGACAGCCTGAGCAGC     2820
Moderna        AAGCTGATCGCCAACCAGTTCAACAGCGCCATCGGCAAGATCCAGGACAGCCTGAGCAGC     2820
Optimized      AAGCTGATCGCCAATCAGTTTAACTCCGCCATCGGCAAGATCCAGGACTCTCTGAGCTCC     2820
               ************** ***** ***  **********************   ******  *

Pfizer         ACAGCAAGCGCCCTGGGAAAGCTGCAGGACGTGGTCAACCAGAATGCCCAGGCACTGAAC     2880
Moderna        ACCGCTAGCGCCCTGGGCAAGCTGCAGGACGTGGTGAACCAGAACGCCCAGGCCCTGAAC     2880
Optimized      ACAGCAAGCGCCCTGGGCAAGCTGCAGGATGTGGTGAATCAGAACGCCCAGGCCCTGAAT     2880
               ** ** *********** *********** ***** ** ***** ******** *****

Pfizer         ACCCTGGTCAAGCAGCTGTCCTCCAACTTCGGCGCCATCAGCTCTGTGCTGAACGATATC     2940
Moderna        ACCCTGGTGAAGCAGCTGAGCAGCAACTTCGGCGCCATCAGCAGCGTGCTGAACGACATC     2940
Optimized      ACCCTGGTGAAGCAGCTGTCTAGCAACTTCGGCGCCATCTCCTCTGTGCTGAATGATATC     2940
               ******** *********     ****************  *   ******** ** ***

Pfizer         CTGAGCAGACTGGACCCTCCTGAGGCCGAGGTGCAGATCGACAGACTGATCACAGGCAGA     3000
Moderna        CTGAGCCGGCTGGACCCTCCCGAGGCCGAGGTGCAGATCGACCGGCTGATCACTGGCCGG     3000
Optimized      CTGAGCAGGCTGGACAAGGTGGAGGCAGAGGTGCAGATCGACCGGCTGATCACAGGCAGA     3000
               ****** * ******      ***** *************** * ******** *** *

Pfizer         CTGCAGAGCCTCCAGACATACGTGACCCAGCAGCTGATCAGAGCCGCCGAGATTAGAGCC     3060
Moderna        CTGCAGAGCCTGCAGACCTACGTGACCCAGCAGCTGATCCGGGCCGCCGAGATTCGGGCC     3060
Optimized      CTGCAGTCCCTGCAGACCTACGTGACACAGCAGCTGATCAGGGCAGCAGAGATCAGGGCA     3060
               ******  *** ***** ******** ************ * ** ** *****  * **

Pfizer         TCTGCCAATCTGGCCGCCACCAAGATGTCTGAGTGTGTGCTGGGCCAGAGCAAGAGAGTG     3120
Moderna        AGCGCCAACCTGGCCGCCACCAAGATGAGCGAGTGCGTGCTGGGCCAGAGCAAGCGGGTG     3120
Optimized      TCTGCCAATCTGGCCGCCACCAAGATGAGCGAGTGCGTGCTGGGCCAGTCCAAGAGAGTG     3120
                  ***** ******************   ***** ************  **** * ***

Pfizer         GACTTTTGCGGCAAGGGCTACCACCTGATGAGCTTCCCTCAGTCTGCCCCTCACGGCGTG     3180
Moderna        GACTTCTGCGGCAAGGGCTACCACCTGATGAGCTTTCCCCAGAGCGCACCCCACGGAGTG     3180
Optimized      GACTTTTGTGGCAAGGGCTATCACCTGATGAGCTTCCCACAGTCCGCCCCTCACGGAGTG     3180
               ***** ** *********** ************** ** ***   ** ** ***** ***

Pfizer         GTGTTTCTGCACGTGACATATGTGCCCGCTCAAGAGAAGAATTTCACCACCGCTCCAGCC     3240
Moderna        GTGTTCCTGCACGTGACCTACGTGCCCGCCCAGGAGAAGAACTTCACCACCGCCCCAGCC     3240
Optimized      GTGTTTCTGCACGTGACCTACGTGCCAGCCCAGGAGAAGAACTTCACCACAGCACCAGCA     3240
               ***** *********** ** ***** ** ** ******** ******** ** *****

Pfizer         ATCTGCCACGACGGCAAAGCCCACTTTCCTAGAGAAGGCGTGTTCGTGTCCAACGGCACC     3300
Moderna        ATCTGCCACGACGGCAAGGCCCACTTTCCCCGGGAGGGCGTGTTCGTGAGCAACGGCACC     3300
```

```
Optimized      ATCTGCCACGATGGCAAGGCACACTTTCCTAGGGAGGGCGTGTTCGTGAGCAACGGCACC     3300
               *********** ***** ** ********  * ** ************  **********

Pfizer         CATTGGTTCGTGACACAGCGGAACTTCTACGAGCCCCAGATCATCACCACCGACAACACC     3360
Moderna        CACTGGTTCGTGACCCAGCGGAACTTCTACGAGCCCCAGATCATCACCACCGACAACACC     3360
Optimized      CACTGGTTTGTGACACAGCGCAATTTCTACGAGCCACAGATCATCACCACAGACAATACA     3360
               ** ***** ***** ***** ** *********** ************* ***** **

Pfizer         TTCGTGTCTGGCAACTGCGACGTCGTGATCGGCATTGTGAACAATACCGTGTACGACCCT     3420
Moderna        TTCGTGAGCGGCAACTGCGACGTGGTGATCGGCATCGTGAACAACACCGTGTACGATCCC     3420
Optimized      TTCGTGTCCGGCAACTGTGACGTGGTCATCGGCATCGTGAACAATACCGTGTATGATCCT     3420
               ******   ******** ***** ** ******** ******** ******** ** **

Pfizer         CTGCAGCCCGAGCTGGACAGCTTCAAAGAGGAACTGGACAAGTACTTTAAGAACCACACA     3480
Moderna        CTGCAGCCCGAGCTGGACAGCTTCAAGGAGGAGCTGGACAAGTACTTCAAGAATCACACC     3480
Optimized      CTGCAGCCAGAGCTGGACTCTTTTAAGGAGGAGCTGGATAAGTACTTCAAGAATCACACC     3480
               ******** *********   ** ** ***** ***** ******** ***** *****

Pfizer         AGCCCCGACGTGGACCTGGGCGATATCAGCGGAATCAATGCCAGCGTCGTGAACATCCAG     3540
Moderna        AGCCCCGACGTGGACCTGGGCGACATCAGCGGCATCAACGCCAGCGTGGTGAACATCCAG     3540
Optimized      AGCCCCGACGTGGATCTGGGCGACATCTCTGGCATCAATGCCAGCGTGGTGAACATCCAG     3540
               ************** ******** ***   ** ***** ******** ************

Pfizer         AAAGAGATCGACCGGCTGAACGAGGTGGCCAAGAATCTGAACGAGAGCCTGATCGACCTG     3600
Moderna        AAGGAGATCGATCGGCTGAACGAGGTGGCCAAGAACCTGAACGAGAGCCTGATCGACCTG     3600
Optimized      AAGGAGATCGACAGGCTGAACGAGGTGGCCAAGAATCTGAACGAGTCCCTGATCGATCTG     3600
               ** ********  ********************* ********  ********* ***

Pfizer         CAAGAACTGGGGAAGTACGAGCAGTACATCAAGTGGCCCTGGTACATCTGGCTGGGCTTT     3660
Moderna        CAGGAGCTGGGCAAGTACGAGCAGTACATCAAGTGGCCCTGGTACATCTGGCTGGGCTTC     3660
Optimized      CAGGAGCTGGGCAAGTATGAGCAGTACATCAAGTGGCCCTGGTATATCTGGCTGGGCTTC     3660
               ** ** ***** ***** ************************* **************

Pfizer         ATCGCCGGACTGATTGCCATCGTGATGGTCACAATCATGCTGTGTTGCATGACCAGCTGC     3720
Moderna        ATCGCCGGCCTGATCGCCATCGTGATGGTGACCATCATGCTGTGCTGCATGACCAGCTGC     3720
Optimized      ATCGCCGGCCTGATCGCCATCGTGATGGTGACCATCATGCTGTGCTGTATGACAAGCTGC     3720
               ******** ***** ************** ** *********** ** ***** ******

Pfizer         TGTAGCTGCCTGAAGGGCTGTTGTAGCTGTGGCAGCTGCTGCAAGTTCGACGAGGACGAT     3780
Moderna        TGCAGCTGCCTGAAGGGCTGTTGCAGCTGCGGCAGCTGCTGCAAGTTCGACGAGGACGAC     3780
Optimized      TGTTCCTGCCTGAAGGGCTGCTGTTCTTGTGGCAGCTGCTGTAAGTTTGATGAGGACGAT     3780
               **   *************** **    ** *********** ***** ** ********

Pfizer         TCTGAGCCCGTGCTGAAGGGCGTGAAACTGCACTACACATGA       3822
Moderna        AGCGAGCCCGTGCTGAAGGGCGTGAAGCTGCACTACACCTGA       3822
Optimized      AGCGAGCCTGTGCTGAAGGGCGTGAAGCTGCACTACACCTAA       3822
                  ***** ***************** *********** * *
```

**Supplementary Figure S1. SARS-CoV-2 Wuhan-Hu-1 spike gene open reading frame comparison.** **A.** Nucleotide identity between original isolate SARS-CoV-2 Wuhan-Hu-1 spike gene sequence (Original) and codon optimized spike gene sequence of SARS-CoV-2 Wuhan-Hu-1 used in this study (Optimized). **B.** Nucleotide identity between spike gene sequence used in Pfizer/BioNtech BNT162b2 (Pfizer) [1], Moderna mRNA-1273 (Moderna) [2] and codon optimized spike gene sequence of SARS-CoV-2 Wuhan-Hu-1 used in this study (Optimized). There is 90.5% nucleotide identity between Pfizer and Moderna sequences, 86.7% identity between Pfizer sequence and optimized sequence used in this study, 87.6% identity between Moderna sequence and optimized sequence used in this study.

Supplementary Figure S2

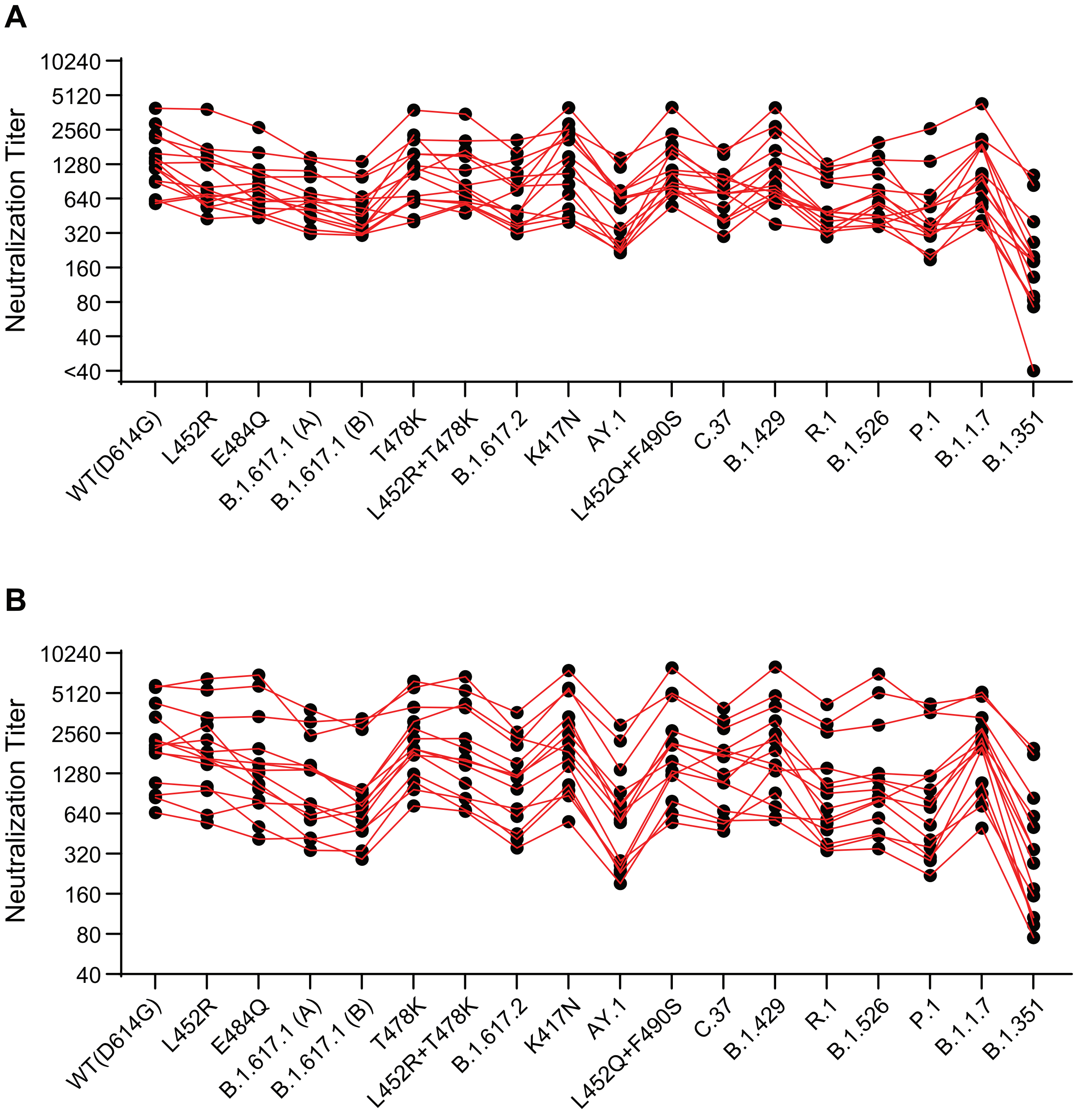


**Supplementary Figure S2. Neutralization of variant SARS-CoV-2 pseudoviruses by vaccination sera.** (**A**) Individual neutralization titers of Pfizer/BioNtech BNT162b2 vaccination sera are presented. (**B**) Individual neutralization titers of Moderna mRNA-1273 vaccination sera are presented. Lines connect responses from a given subject against the indicated variants.

Supplementary Figure S3

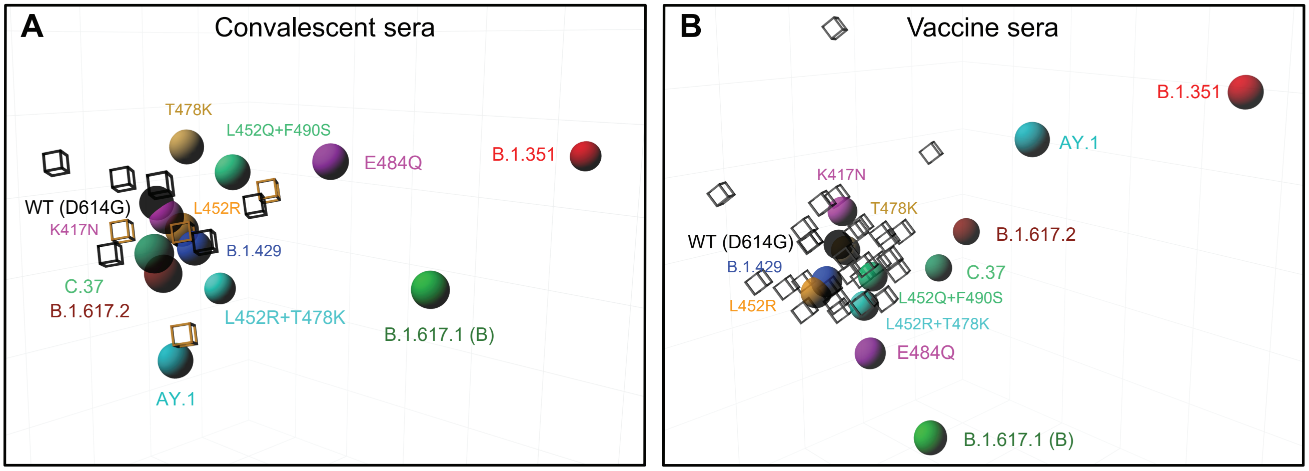


**Supplementary Figure S3. 3D antigenic maps of SARS-CoV-2 variants made using antigenic cartography**. Antigenic maps were made using neutralizing antibody titers from convalescent sera (A) and vaccine sera (B). Convalescent sera map (A) includes sera from the group of WT(D614G)-infected individuals and the group of individuals infected with strains containing L452R mutation. Vaccine sera map (B) includes sera from individuals vaccinated with Pfizer/BioNtech BNT162b2 and individuals vaccinated with Moderna mRNA-1273 vaccine. Sera are shown as open squares, pseudoviruses are shown as colored circles, labeled by strain name.  Each grid-square corresponds to a two-fold dilution in the pseudovirus neutralization assay. Antigenic distance is interpretable in any direction.

Supplementary Figure S4

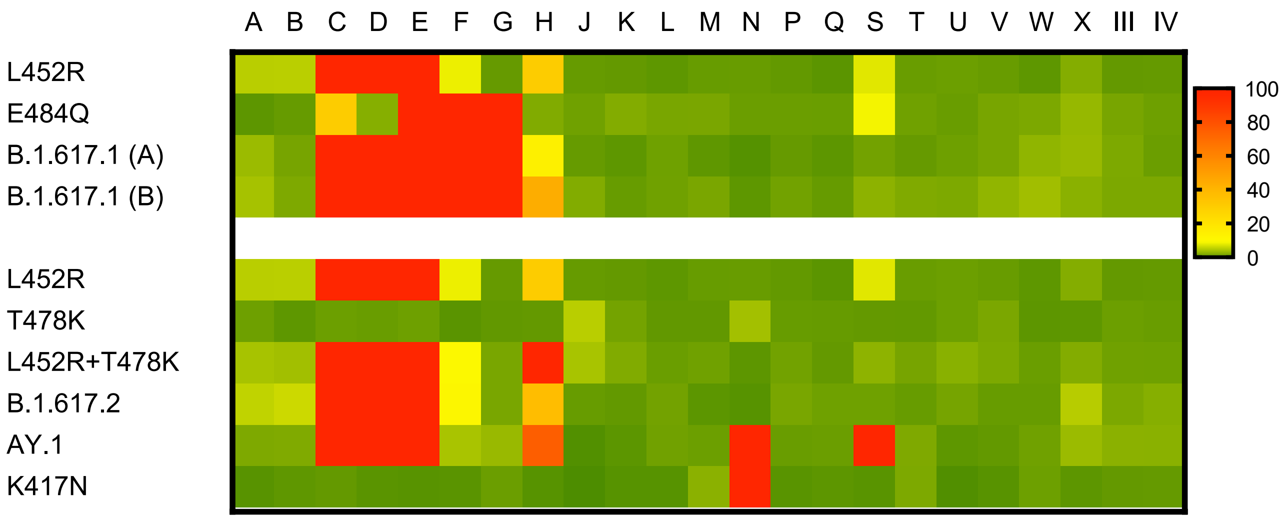


**Supplementary Figure S4. Neutralization of variant SARS-CoV-2 pseudoviruses by therapeutic antibodies.** The antibody panel, consisting of 15 single nAbs, 6 cnAbs, and 2 pAbs were tested against pseudoviruses bearing variant spikes and spikes with indicated RBD substitutions in the D614G background. Antibodies are blinded-coded according to an agreement with the manufacturers. Heat map representing the ratio of $IC_{50}$ values of the variant or RBD mutant spike relative to WT(D614G). Red indicates loss of potency ($IC_{50}$ ratios > 50). Yellow indicates moderate loss of potency ($IC_{50}$ ratios between 10-50), and green indicates retention of potency ($IC_{50}$ ratios <10).

Supplementary Figure S5

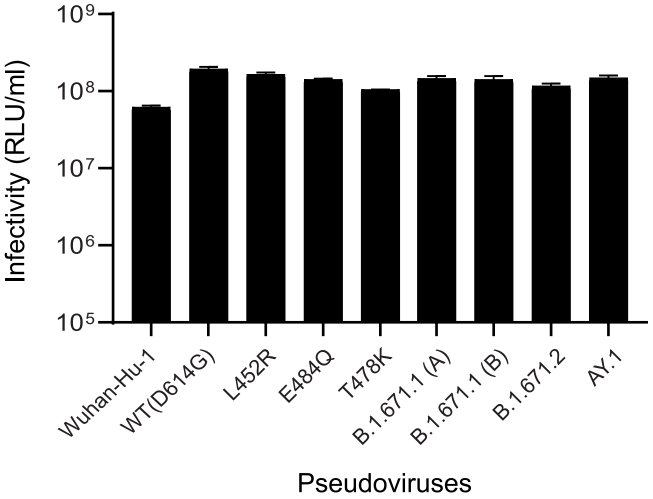


**Supplementary Figure S5. Infectivity of pseudoviruses with variants spikes.** Spike-bearing pseudoviruses infectivity in 293T-ACE2.TMPRSS2 cells. Cells were infected with pseudoviruses bearing spikes from SARS-CoV-2 variants. Bars: Mean +/- SD.

Supplementary Figure S6

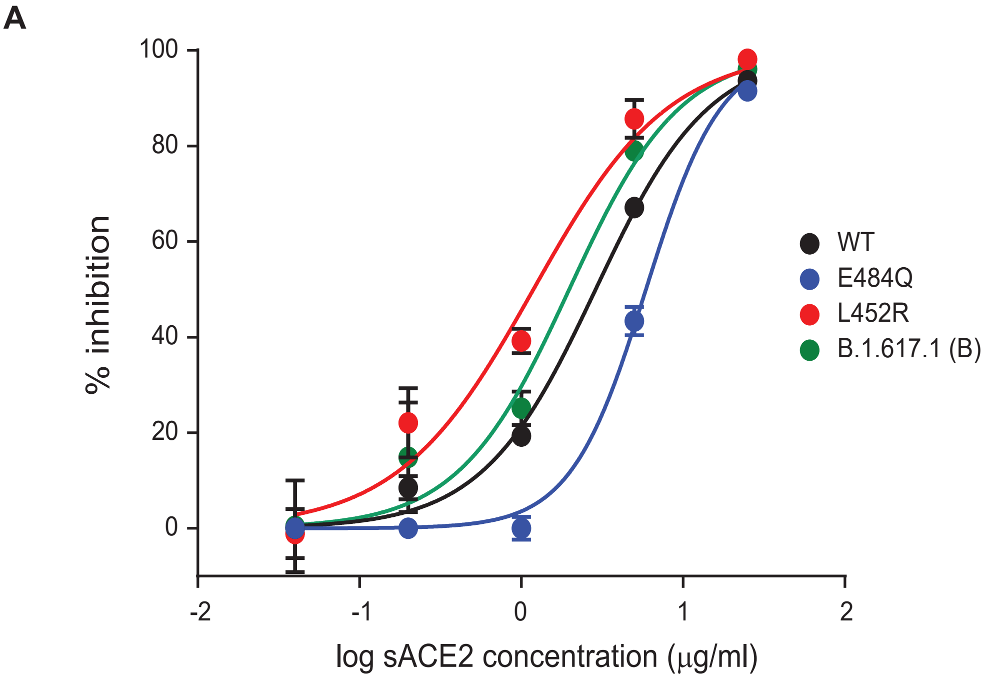


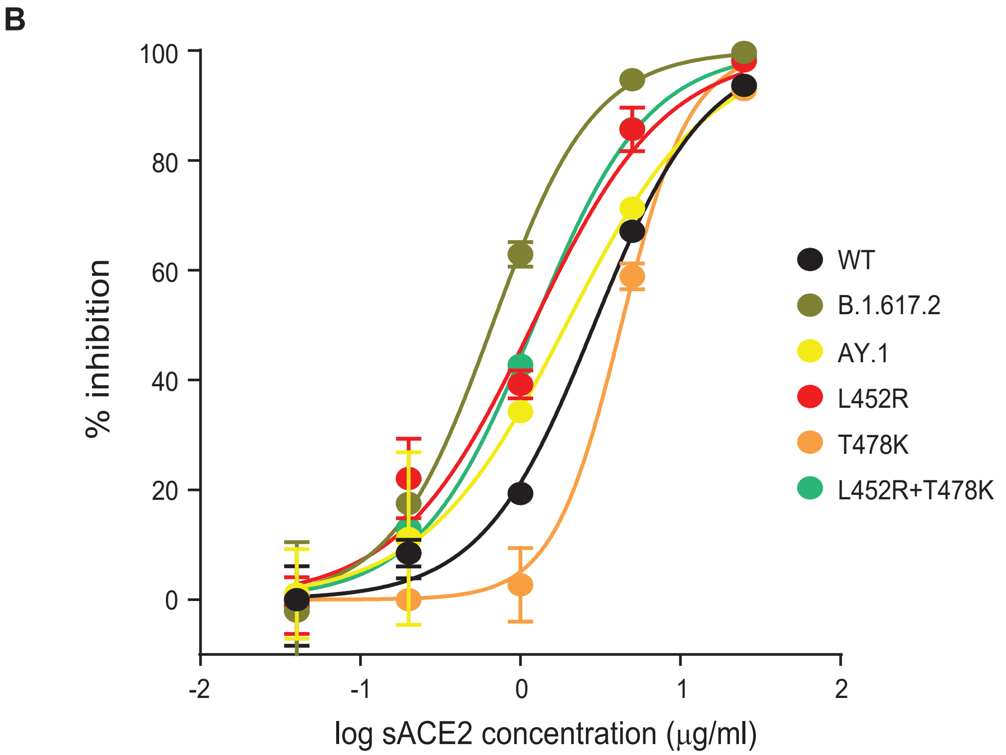


**Supplementary Figure S6. Neutralization of B.1.617 variant pseudoviruses by soluble ACE2.** Pseudoviruses bearing B.1.617.1 (**A**) and B.1.617.2 (**B**) variant spike proteins were incubated with a serially diluted soluble ACE2 (sACE2) and then applied to 293T-ACE2.TMPRSS2 cells for infection. Each curve indicates percent inhibition of pseudoviruses by sACE2. The image represents results of one of three independent experiments.

**Supplementary Table S1.** Dimensionality testing of Antigenic Cartography maps

| Dimension | Convalescent sera map | | | | Vaccine sera map | | | |
|---|---|---|---|---|---|---|---|---|
| | Measured titers | | Thresholded titers | | Measured titers | | Thresholded titers | |
| | Mean RMSE | variance RMSE | Mean RMSE | variance RMSE | Mean RMSE | variance RMSE | Mean RMSE | variance RMSE |
| 1D | 0.94 | 0.02 | 1.26 | 0.37 | 0.54 | 0.003 | 2.38 | 0.06 |
| 2D | 0.95 | 0.15 | 1.41 | 1.18 | 0.51 | 0.003 | 2.18 | 0.07 |
| 3D | 0.89 | 0.12 | 1.36 | 1.11 | 0.49 | 0.003 | 2.26 | 0.03 |
| 4D | 0.88 | 0.14 | 1.34 | 1.14 | 0.49 | 0.002 | 2.23 | 0.02 |
| 5D | 0.91 | 0.24 | 1.41 | 1.73 | 0.49 | 0.002 | 2.23 | 0.02 |

Dimensionality testing to identify best number of dimensions for fitting the antigenic maps. Each dataset was tested with cross-validation in 1-5 dimensions (100 maps, each made with 75% of the data to predict the exclude 25%), with low mean and variance in root-mean squared error (RMSE) for measured titers (within limit of detection) and thresholded titers (below the limit of detection) indicating the optimal number of dimensions. Convalescent sera from individuals infected either WT(D614G) or strains with L452R mutation. Vaccine sera from individuals vaccinated with either Pfizer/BioNtech BNT162b2 or Moderna mRNA-1273 vaccine.

**References:**


1. World Health Organization. Messenger RNA Encoding the Full-Length SARS-CoV-2 Spike Glycoprotein. 2020. Available online: https://web.archive.org/web/20210105162941/https://mednet-communities.net/inn/db/media/docs/11889.doc (accessed on 30 November 2021).
2. Jeong, D. E.; McCoy, M.; Artiles, K.; Ilbay, O.; Fire, A.; Nadeau, K.; Park, H.; Betts, B.; Boyd, S.; Hoh, R.; et al. Assemblies-of-Putative-SARS-CoV2-Spike-Encoding-mRNA-Sequences-for-Vaccines-BNT-162b2-and-mRNA-1273. Available online: https://virological.org/t/assemblies-of-putative-sars-cov2-spike-encoding-mrna-sequences-for-vaccines-bnt-162b2-and-mrna-1273/663 (accessed on 30 November 2021).
